# Gastrointestinal, metabolic, and nutritional disorders: A plant-based ethnoveterinary meta-analysis in the Catalan linguistic area

**DOI:** 10.3389/fvets.2022.908491

**Published:** 2022-08-09

**Authors:** Fuencisla Cáceres, Joan Vallès, Teresa Garnatje, Montse Parada, Airy Gras

**Affiliations:** ^1^Laboratori de Botànica - Unitat Associada CSIC, Facultat de Farmàcia i Ciències de l'Alimentació - Institut de Recerca de la Biodiversitat IRBio, Universitat de Barcelona, Barcelona, Spain; ^2^Secció de Ciències Biològiques, Institut d'Estudis Catalans, Barcelona, Spain; ^3^Institut Botànic de Barcelona (IBB), CSIC-Ajuntament de Barcelona, Barcelona, Spain; ^4^Center for the Study of Human Health, Emory University, Atlanta, GA, United States

**Keywords:** Catalan linguistic area, ethnoveterinary, gastrointestinal disorders, Iberian Peninsula, medicinal plants, traditional knowledge

## Abstract

Veterinary care is fundamental for animal wellbeing, and so is achieving a comprehensive understanding of traditional ethnoveterinary applications. However, little attention has been paid to it so far in industrialized countries, and in particular in Western Europe. In this context, the present work aims to make a contribution to this issue in the Catalan linguistic area, focusing on the study of plants used, at a popular level, to treat and deal with gastrointestinal, metabolic, and nutritional disorders, which are among the most important issues that affect animals. Data obtained in this study come from the popular knowledge about plants for veterinary purposes from 599 informants, who jointly provided 1,405 reports of use from 148 plant taxa. The most cited species have been *Tanacetum parthenium* (L.) Sch.Bip. (9.04%), *Olea europaea* L. subsp. *europaea* var. *europaea* (6.26%), and *Euphorbia lathyris* L. (6.26%). At higher taxonomic levels, the botanical families with more ethnoveterinary applications were Asteraceae (24.48%), Euphorbiaceae (8.33%), and Oleaceae (7.12%). Among the total use reports, 95.02% refer to disorders of the gastrointestinal system, 4.34% to nutritional disorders, and 0.64% to metabolic disorders. Antidiarrheal (18.01%), digestive (16.51%), and laxative (15.80%) have been the most reported veterinary uses. The most used plant parts have been the aerial part (40.50%), the fruit or the infructescence (18.65%), and the flower or inflorescence (16.01%). The main preparation and administration forms reported were tisane (58.69%), followed by direct use (without any specific pharmaceutical form; 21.77%). The global corpus of ethnoveterinary knowledge for the gastrointestinal system disorders in the territory of study is diverse, with some species having a very high cultural value, as indicated by an informant consensus factor very close to 1. Some reported uses were also confirmed after consultation of encyclopedic pharmacological works, although few of these works are specifically devoted to veterinary uses. The results of this study are relevant to preserve the ethnoveterinary knowledge, but also represent an important contribution to be taken into account in research for future development of new plant-based drugs for animals.

## Introduction

Medicinal plants have been used extensively worldwide and throughout history to treat and prevent the occurrence of various diseases, infections, and infestations in domestic animals, mainly in livestock (1). Likewise, traditional veterinary practices have been documented for more than 14,000 years and they are as ancient as the domestication of animals (2, 3). To provide experimental evidence, in recent years, traditional uses of many medicinal plants have been tested under laboratory conditions (4).

Gastrointestinal disorders have been the principal cause of animal mortality, delays in weight gain, and consequently loss in productivity of livestock. Based on that, it is, therefore, crucial to focus on the underpinning causes of illnesses and disorders, aiming at improving and maintaining the profitability of these animals and the services they produce to the human population (5). In addition, other disorders of metabolic and nutritional nature also affect drastically their maximum potential as well as the animals' survival, but their prevalence and impact compared to gastrointestinal ones are, in general, lower (6).

Ethnoveterinary knowledge (EVK) refers to knowledge, skills, practices, and folk beliefs in relation to animal care, which human beings use and have mostly domesticated throughout history (7). Ethnoveterinary medicine (EVM), in particular, is the scientific term used to refer to the tradition of guaranteeing animal health. Ethnoveterinary medicine is based on popular knowledge about diseases affecting animals, and how to manage them, based on clinical remedies and practices for the treatment and prevention of veterinary diseases, and establishing strategies and spiritual elements related to animal welfare and their production (8). Frequently, EVM is based on medicinal plants, not rarely wild, but also relies on the utilization of products of animal and mineral origin (8), and procedures such as bone fixations and vaccinations. In any case, EVK inevitably requires the component of human intervention (7).

In several research studies, it is possible to find documented evidence about the medicinal role of plant secondary metabolites, which have been of great interest to researchers worldwide as alternatives or complementary drugs to synthetic agents (9, 10). In contrast, investigation in the field of ethnoveterinary medicine is limited compared to our current understanding of traditional remedies to treat human diseases, and further prospection in this field is, therefore, needed (11). Certainly, back in the 2000s, the overarching theme of the 10th edition of the International Congress of the International Society of Ethnopharmacology was entirely focused on different topics of ethnoveterinary (1).

Until now, much of the research carried out in the field of ethnoveterinary has been implemented in Africa and Asia (12–16), although there is an increasing interest in this field in Europe (17–20). In Spain, for example, the growing interest in ethnoveterinary is illustrated by the substantial number of studies and research published in recent years (8, 21–24). In addition, as mentioned above and due to the significant impact of gastrointestinal disorders on veterinary, this issue has become the principal focus of study in Croatia (25), West Africa (26), and Congo (27).

The Catalan linguistic area (CLA) is one of the most largely studied territories in Europe from the ethnobotanical point of view (28). In this region, some ethnoveterinary studies based on small geographical areas have been published (29, 30), but a large-scale meta-analysis has never been addressed. Based on that, the main objectives of this study were: (i) to report uses of plants to treat gastrointestinal, metabolic, and nutritional disorders in ethnoveterinary, based on information of traditional uses recorded in CLA; (ii) to conduct a comprehensive literature review of pharmacological activity and uses of plants concerned; and (iii) to provide pieces of evidence regarding for the importance of traditional knowledge as a resource in veterinary research for future development of new plant-based drugs for animal health.

## Materials and methods

### Study area

Catalan linguistic area, including the Catalan-speaking area, Catalan language territories, and Catalan countries, constitutes a well-studied unit under different approaches: geographic (31), physiographic (32), floristic (33, 34), vegetation (35), and linguistic and cultural (36). This territory, mostly located in the eastern part of the Iberian Peninsula, also includes a northern Pyrenean portion, the Balearic Islands, and the city of L'Alguer on the island of Sardinia. Politically, these territories, with an extension of 70,000 km^2^ (34), and ~14,000,000 inhabitants (37–41), belong to four states: Andorra (all the territory), France (Northern Catalonia or Eastern Pyrenees department), Italy (L'Alguer, Sardinia), and Spain (Balearic Islands, Carxe—a small area in Murcia, Catalonia, a portion of eastern Aragon, and Valencia). This territory spans from the Mediterranean Sea level to 3,143 m a.s.l. in Pica d'Estats (Pyrenees). The diversity of landscapes available is also variable, and structured in several stages with distinct floristic and vegetation traits (34, 35), harboring ~4,300 autochthonous and 1,200 allochthonous plant taxa, including species and subspecies^1^.

### Databasing and data selection

All the information gathered has been collected through semi-structured ethnobotanical interviews (42), following the ethical principles of the International Society of Ethnobiology (43), and included in an open-access webpage (https://etnobotanica.iec.cat), which contains the ethnobotanical data in CLA (44). Herbarium vouchers of plants used and reported by informants are deposited in the herbarium BCN (Center de Documentació de Biodiversitat Vegetal, Universitat de Barcelona).

**Figure 1:**
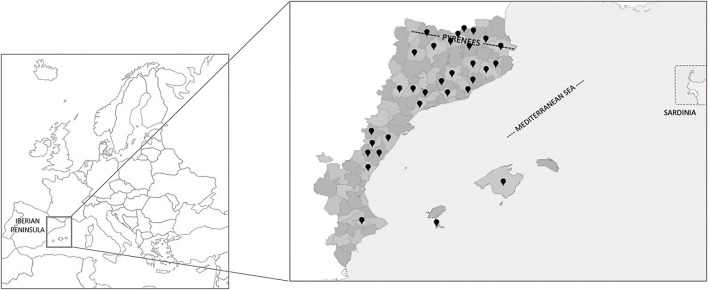
Geographical map of the territories studied within Europe in the Catalan linguistic area (NE Iberian Peninsula). Black pointed dots indicate the specific territories surveyed and analyzed.

The information concerning ethnoveterinary data to treat gastrointestinal, metabolic, and nutritional disorders has been obtained from the open access webpage mentioned above, and the study area covers 31 territories [29 districts, namely, “comarca” (i.e., comparable to counties) in the Catalan language, plus Mallorca and Formentera islands; Figure 1]. A minor bias exists because, in one out of the studies included in the dataset (45), each taxon is assigned to a municipality, instead of informants, as is the case in all the remaining works. This can result in a slight underestimation of both usage reports and the indexes that include them.

For taxon nomenclature, Bolòs et al.'s work (34) has been followed, which is a flora covering specifically the area considered, and Plants of the World Online (https://powo.science.kew.org) for the exotic plants. In addition, for botanical family attribution, we have followed the criteria established by the last Angiosperm Phylogeny Group (APG IV (46)).

### Data analysis

The descriptive statistics and quantitative ethnobotany analyses were carried out using Excel (Microsoft Excel 2016). To analyze the results, the use report (hereinafter, UR) value has been used (47). Moreover, to assess the state of knowledge, some ethnobotanical indices were also applied: (1) the ethnobotanicity index (EI) (48), which is the quotient between the number of plant taxa used (here taking into account the plants used to treat gastrointestinal, metabolic, and nutritional disorders in veterinary), by the total number of plant taxa that constitute the flora of the territory (autochthonous plants), expressed as a percentage, to have a general idea of the relevance of these plants in the area considered; (2) informant consensus factor (F_IC_) (49), which is the ratio between the number of UR minus the number of plant taxa used, and the number of UR minus one. This value is calculated to assess the consistency or robustness of the traditional knowledge regarding gastrointestinal, metabolic, and nutritional disorders in the territory; (3) the cultural importance index (CI) (50), which is represented by the sum of the proportion of informants that mention each taxon use, has also been calculated to identify the plants most valued by the informants; and (4) the medicinal importance index (MI) (51), the quotient between the total UR for a specific use category and the number of plant taxa possessing this use, to evaluate the real importance of the use.

### Results comparison in phytotherapy and pharmacology sources

A review of pharmacological sources was performed to acquire the number of taxa that have been previously studied. The pharmacological comparison has been performed using human and veterinary monographs from official sources and encyclopedic bibliography on phytotherapy (52–57). The lack of exclusive monographs and encyclopedic bibliography in veterinary has conditioned us to use human sources as well.

## Results and discussion

### General data

The number of use reports extracted from the dataset of gastrointestinal, metabolic, and nutritional disorders analyzed was 1,405, and this information came from 599 informants (Supplementary material S1).

A total of 148 taxa were cited in the surveys carried out in CLA. Two out of the total number of taxa are determined only at a generic level, and 20 at an infraspecific level. The comparison between the data presented in this study with general ethnoveterinary studies developed around the world showed that the number of taxa reported in the present study for a specific group of diseases is higher than the total number of ethnoveterinary plants in other regions of the Iberian Peninsula, that is, Huesca (78 taxa) (22), Navarra (36 taxa) (8), Castilla y León (84 taxa) (23), and Castilla-La Mancha (83) (24). Likewise, the same trend was recovered when considering other territories around the world such as Italy (42 taxa) (18), Algeria (66 taxa) (12), Pakistan (56 taxa) (16), Uganda (50 taxa) (7), Namibia (15 taxa) (13), or China (39 taxa) (14), except for Palestine (138 taxa) (58). Concerning works focusing on the same kind of illnesses addressed in the present article, our results were only slightly lower than those (158 taxa) from West Africa (26), and largely higher than the nine taxa reported from another African territory (Congo (27)) and a European one (Croatia (25)).

The average of use reports per informant was 2.35, although 52.92% of informants only reported one veterinary use of this group of disorders. This percentage indicates that more than half of the informants know very few plants that can be used to cure/treat animals. Another possible fact to explain this scenario could be that many of the informants have a limited number of domestic animals, for example, for familiar use. Probably shepherds, and particularly those having practiced transhumance in the past, could have faced more challenges related to animal health issues and would have reported potentially more abundant information. Nevertheless, Rivera et al. (24) studied transhumance-associated ethnoveterinary practices in a large area (Castilla-La Mancha, Spain) and found only 83 plant species and 676 reports for all medicinal uses addressed to animals. Among them, only 12.3% of the reports were related to the treatment of gastrointestinal issues. In addition, a possible bias could be because during the interviews more questions are addressed to the medicinal plants than to the veterinary ones. However, irrespective of the relatively small pool of ethnoveterinary plants recorded in the area considered, it is worth mentioning that the informant consensus factor (F_IC_) calculated to assess the state of ethnoveterinary knowledge about the plants used to treat gastrointestinal, metabolic, and nutritional disorders in CLA and the value obtained was relatively high, F_IC_ = 0.90 (this parameter ranges from 0 to 1). This indicator reveals a strong agreement or consensus in relation to the information obtained for the plants used in the studied area, accounting for the consistency and robustness of the information corpus obtained. The ethnobotanicity index (EI), a parameter estimated only taking into account the autochthonous taxa recorded (those included in Bolòs et al. (34)), was relatively low for ethnoveterinary uses in the studied area, EI = 3.35%. Unfortunately, as far as we are aware, the EI value is not available in other territories for ethnoveterinary uses, which prevents us from making direct comparisons. That said, the value obtained in our study is lower than that for general human medicinal studies (14.44%) obtained from surveys carried out in the same area (59). Similarly, other specific medicinal disorders, such as those to treat infections, also produced higher EI (7.26%) than that obtained for ethnoveterinary uses (60). Overall, such a trend is somehow expectable based on the fact that, in general, fewer data are recorded on medicinal plant uses addressed to treat animals than they are for humans.

In general, the global corpus of ethnoveterinary knowledge gathered in this study is diverse and rich, based on the non-negligible absolute number of use reports and the high values of some of the above-mentioned indices. However, we must acknowledge the existence of potential drawbacks, such as those associated with the substantial loss of traditional plant knowledge and the decline of local, folk veterinary uses. Without doubts, we live a present driven by the loss of traditional livestock-raising activities, which are outcompeted by the continuous development of modern intensive animal farms. The latter, mostly rely on the application of standard veterinary medication practices, so the urge for inventories of extant traditional knowledge in this field is more necessary than ever, before such cultural heritage is lost for good (8).

### Taxa and parts of plants used

Based on our surveys, the 148 plant taxa with records of EVM use are distributed among 56 botanical families. The most cited families, which represent more than half of total use reports (50.60%) were Asteraceae (24.48%, 16 taxa), Euphorbiaceae (8.33%, three taxa), Oleaceae (7.12%, two taxa), Malvaceae (5.84%, four taxa), and Fagaceae (4.84%, four taxa). It is not surprising that the Asteraceae family accounts for the largest in the number of use reports and diversity of taxa, as it is one of the most diverse and speciose families in the Mediterranean flora and around the world (61). However, the remaining most quoted families (Euphorbiaceae, Oleaceae, Malvaceae, and Fagaceae), were not among the most highly reported in general ethnobotanical studies. It is worth mentioning that these families are represented in this study by a small number of taxa per family, which indicates a high specificity in terms of used plants. In Malvaceae, for example, only four species were reported and 78.05% of the family's use reports were attributed to only one species, *Malva sylvestris* L. Another interesting point worth highlighting refers to the contrasting rates of use and applications at the family level between territories. For example, the Euphorbiaceae family is reported in West Africa among the most quoted to treat gastrointestinal disorders (26), but in CLA, reports of use mostly came associated with its laxative and purgative effects. Fabaceae (3.35%, 13 taxa) and Rosaceae (2.42%, 10 taxa) are also relatively large families, following Asteraceae in terms of diversity of taxa. However, when it comes to percentages of the total use reports, their incidence is low, indicating that there is no direct relationship between the species diversity and rates of use.

Regarding the type of disorders, the most used taxa to treat gastrointestinal, metabolic, and nutritional disorders in veterinary are summarized in Table 1. Among the most quoted species, we can find *Tanacetum parthenium* (L.) Sch.Bip. (9.04%), *Euphorbia lathyris* L. (6.26%), *Olea europaea* L. subsp. *europaea* var. *europaea* (6.26%), *Tanacetum vulgare* L. (5.55%), and *M. sylvestris* (4.56%). Comparing the most used taxa in our area of study, with another work carried out in Croatia to treat indigestions and diarrhea (25), the results showed that three of the nine taxa quoted in the Balkan area are also listed among those most mentioned in CLA (Table 1). The species shared between these two ethnobotanical inventories are *Achillea millefolium* L., *Linum usitatissimum* L., and *M. sylvestris*.

**Table 1:** The most cited plants to treat gastrointestinal, metabolic, and nutritional disorders in veterinary, with the most common vernacular names, the veterinary uses, the number of total use reports and percentage, and the CI index.

**Taxon (family) and herbarium voucher**	**Local name (in Catalan language)**	**Veterinary use**	**Total UR**	**Total UR (%)**	**CI index**
*Tanacetum parthenium* (L.) Sch.Bip. (Asteraceae) BCN 25014	Camamilla, camamilla amarga, camamilla borda	Antidiarrheal, anti-icteric, digestive, emetic, laxative, ruminant antistatic	127	9.04	0.21
*Euphorbia lathyris* L. (Euphorbiaceae) BCN 24884	Cagamuja, herba talpera	Emetic^1^, purgative^1^, ^5^	88	6.26	0.15
*Olea europaea* L. subsp. *europaea* var. *europaea* (Oleaceae) BCN 125505	Olivera, oliver	Antidiarrheal, buccal antiseptic, carminative, emetic, intestinal anti-inflammatory, laxative^1^, ^4^, orexigenic, purgative, ruminant antistatic	88	6.26	0.15
*Tanacetum vulgare* L. (Asteraceae) BCN 29803	Comí marrà, herba cuquera, tanarida	Antidiarrheal, digestive, intestinal anti-inflammatory, laxative, orexigenic, purgative, ruminant antistatic	78	5.55	0.13
*Malva sylvestris* L. (Malvaceae) BCN 125508	Malva, mauva, vauma	Antidiarrheal, buccal antiseptic^1^, digestive^1^, ^2^, ^5^, ^5^, gastric anti-inflammatory^1^, ^3^, ^4^, laxative^1^, ^4^, purgative, stomachic	64	4.56	0.11
*Achillea millefolium* L. (Asteraceae) BCN 125391	Herba de tall, milfulles, milifulla	Antidiarrheal^1^, digestive^2^, ^3^, for colic^1^, ^4^, laxative	52	3.70	0.09
*Rubia peregrina* L. (Rubiaceae) BCN 125499	Herba apegalosa, herba remuguera, herba de remuc	Antidiarrheal, digestive, ruminant antistatic	44	3.13	0.07
*Linum usitatissimum* L. (Linaceae) BCN 47281	Lli, llinet, llinosa	Antidiarrheal^1^, carminative, digestive^2, 4^, laxative^1^, ^2^, ^3^, ^4^, ^6^, ruminant antistatic	41	2.92	0.07
*Quercus ilex* L. (Fagaceae) BCN 125517	Alzina, alzinera, olina	Antidiarrheal^1^, ^4^, carminative, emetic, for gaining weight, for gastrointestinal disorders	41	2.92	0.07
*Santolina chamaecyparissus* L. (Asteraceae) BCN 96763	Camamilla, camamilla de botó, espernallac	Antidiarrheal, buccal antiseptic, digestive^1^, ^4^, gastric anti-inflammatory^4^, hepatoprotective, intestinal anti-inflammatory^4^, stomachic^1^	40	2.85	0.07
*Bryonia cretica* L. subsp. *dioica* (Jacq.) Tutin (Cucurbitaceae) BCN 140164	Carabassina, carabassera borda, tuca	Gallbladder anti-inflammatory, purgative, ruminant antistatic	32	2.28	0.05
*Thymus vulgaris* L. (Lamiaceae) BCN 96764	Farigola, timó, timonet	Antidiarrheal, anti-tympanic, buccal antiseptic^1^, digestive^1^, for digestive disorders, intestinal anti-inflammatory, ruminant antistatic	26	1.85	0.04
*Urtica dioica* L. (Urticaceae) BCN 25030	Estrígol, ortiga, otriga	Digestive, for gaining weight, laxative	25	1.78	0.04
*Helleborus foetidus* L. (Ranunculaceae) BCN 29705	Escampador, manxiula, marxívol	Carminative, intestinal anti-inflammatory	24	1.71	0.04
*Lippia triphylla* (L'Hér.) O.Kuntze (Verbenaceae) BCN 125394	Marialluïsa, herballuïsa	Digestive^4^, gastric anti-inflammatory	24	1.71	0.04
*Ricinus communis* L. (Euphorbiaceae) BCN 46089	Ricí	Laxative^1^, ^2^, ^4^, purgative^1^	24	1.71	0.04
*Daphne gnidium* L. (Thymelaeaceae) BCN 29687	Tei, matapoll	Antidiarrheal	20	1.42	0.03
*Triticum aestivum* L. (Poaceae) BCN 156578	Blat	Antidiarrheal^1^, ^4^, for gastrointestinal disorders^5^, laxative	20	1.42	0.03
*Ceratonia siliqua* L. (Fabaceae) BCN 32177	Garrofer, garrover	Antidiarrheal^1^, ^4^	17	1.21	0.03
*Juniperus communis* L. (Cupressaceae) BCN 29878	Ginebre, ginebró	Antidiarrheal, for gastrointestinal disorders^1^, ^2^, ^4^	17	1.21	0.03

Comparison of uses in pharmacological comprehensive literature:

1 Duke (53),

2 EMA (49),

3 ESCOP (50),

4 Fitoterapia.net (51),

5 Xie's Chinese Veterinary Herbology (54),

6 Blumenthal (52).

UR, use report; CI index, cultural importance index.

While most plants have multiple uses, *E. lathyris*, which represents the second most quoted taxon, has only one specific use. This is certainly, a remarkable fact because, all but one of its 88 UR claim the plant to be laxative (see below for the relation of this fact with the vernacular name of the plant). Possessing such a veterinary property is quite appreciated among informants, since many of the plants reported in this study are indicated for this use (or the similar purgative), including the species *Ricinus communis* L. (also used by humans (62, 63)). Interestingly, the opposite effect, that is, antidiarrheal, is also highly reported, being the taxa *Daphne gnidium* L. and *Ceratonia siliqua* L. exclusively indicated for this purpose.

Unlike human medicinal studies, in which exotic species have been frequently introduced as useful plants (e.g., (42, 64)), in veterinary, the plants cited to treat gastrointestinal, metabolic, and nutritional disorders were mostly autochthonous (see above for the reach of this term concept), except two, *Cassia angustifolia* Vahl., used as a purgative, and *Coffea arabica* L., as a digestive. This fact could be related to those veterinary uses being more neglected than human medicinal uses, as previously mentioned.

Of the 148 taxa reported in this study, nine of them have also been reported to treat gastrointestinal, metabolic, and nutritional disorders in humans: *A. millefolium, E. lathyris, L. usitatissimum, Lippia triphylla* (L'Hér.) O.Kuntze*, M. sylvestris, O. europaea* subsp. *europaea* var. *europaea, Santolina chamaecyparissus* L., *T. parthenium*, and *Thymus vulgaris* L. (65). The relationship between ethnoveterinary and traditional human medicine is reciprocal and to some extent, both tend to co-evolve in parallel. It is not rare that remedies known and used for human wellbeing could be employed as well to treat animals, and likewise, remedies used by animals to self-medicate could later be incorporated and utilized to treat human pathologies (13).

The cultural importance (CI) index is calculated to obtain the degree of appreciation by the informants for the plants they use. This index (Table 1) is coinciding in importance order with the most quoted taxa, and the maximum value obtained was 0.21 for *T. parthenium*. The information provided by the CI index, from a robust dataset (see above the informant consensus factor), about the positive perception of some plants by the informants, has a great value as an indicator to set the initial steps to potentially develop new drugs in veterinary. Another species of the same genus, *T. vulgare*, also appears among the only four plants with a CI index higher than 0.1, and two more taxa from the same family (i.e., Asteraceae: *A. millefolium* and *S. chamaecyparissus*) are classified as well within the 20 top plants (Table 1), illustrating the overall relevance of this family in EVM.

Concerning cultural aspects, it is worth mentioning that some folk Catalan names recorded refer to medicinal uses. In some cases, the name given to the plant is coincidental with the precise ethnoveterinary use. For example, *E. lathyris* is popularly known as “cagamuja” in Catalan, which can be translated into English as “to defecate.” Also, *Rubia peregrina* L., a rumination herb is named “herba remuguera” and/or “herba de remuc” to allude, specifically, to the purgative and ruminal antistatic properties attributed to this plant.

The parts of the plant most commonly used to treat gastrointestinal, metabolic, and nutritional disorders were the aerial structures (40.50%), followed by fruit or infructescence (18.65%), flowers or inflorescences (16.01%), and seeds (7.62%). These results are similar to those reported in other geographical and cultural areas prospected in the Iberian Peninsula (8, 64). Certainly, the predominant use of these parts is, to some extent, intuitive since they correspond to the most visible and accessible plant organs. The same argument is that the closer is to human settlements the more it is locally used (66).

### Veterinary uses and pharmaceutical forms

The list of medicinal uses quoted here were grouped in those addressed to gastrointestinal (95.02%), nutritional (4.34%), and metabolic (0.64%) disorders. In total, 26 veterinary uses were reported (Table 2). Among the most quoted uses we found antidiarrheal (18.01%, 56 taxa), digestive (16.51%, 31 taxa), laxative (15.80%, 36 taxa), purgative (12.17%, 13 taxa), and ruminant antistatic (7.28%, 16 taxa). The highest proportion of uses registered are commonly found in other similar studies (26) and, in general, in ethnoveterinary studies, where gastrointestinal disorders are among the most treated in different parts of the world (8, 12, 14, 20, 24, 58).

**Table 2:** Veterinary uses to treat gastrointestinal, metabolic, and nutritional disorders in veterinary and values of total use reports, total use reports percentage, and medicinal importance index.

**Veterinary use**	**Total UR**	**Total UR (%)**	**Total taxa**	**MI index**
Antidiarrheal	253	18.01	56	4.52
Digestive	232	16.51	31	7.42
Laxative	222	15.80	36	6.14
Purgative	171	12.17	13	13.15
Ruminal antistatic	118	8.40	16	7.38
Intestinal anti-inflammatory	68	4.84	25	2.72
Carminative	65	4.63	9	7.22
For gaining weight	58	4.13	6	9.67
For gastrointestinal disorders	37	2.63	15	2.47
Hepatoprotective	32	2.28	8	4.00
Emetic	31	2.21	7	4.43
Buccal antiseptic	23	1.64	10	2.30
Hepatic anti-inflammatory	20	1.42	6	3.33
Anti-icteric	15	1.07	7	2.14
Orexigenic	12	0.85	6	2.00
Gastric anti-inflammatory	9	0.64	4	2.25
Stomachic	9	0.64	5	1.80
For colic	9	0.64	2	4.50
Diaphoretic	7	0.50	1	7.00
Gallbladder anti-inflammatory	4	0.28	2	2.00
Vitamin	3	0.21	3	1.00
Anti-tympanic	2	0.14	2	1.00
Cooling agent	2	0.14	1	2.00
Gingival anti-inflammatory	1	0.07	1	1.00
Gastric or intestinal emollient	1	0.07	1	1.00
Dental strengthening	1	0.07	1	1.00

UR, use report; Medicinal importance index (MI index).

**Figure 2:**
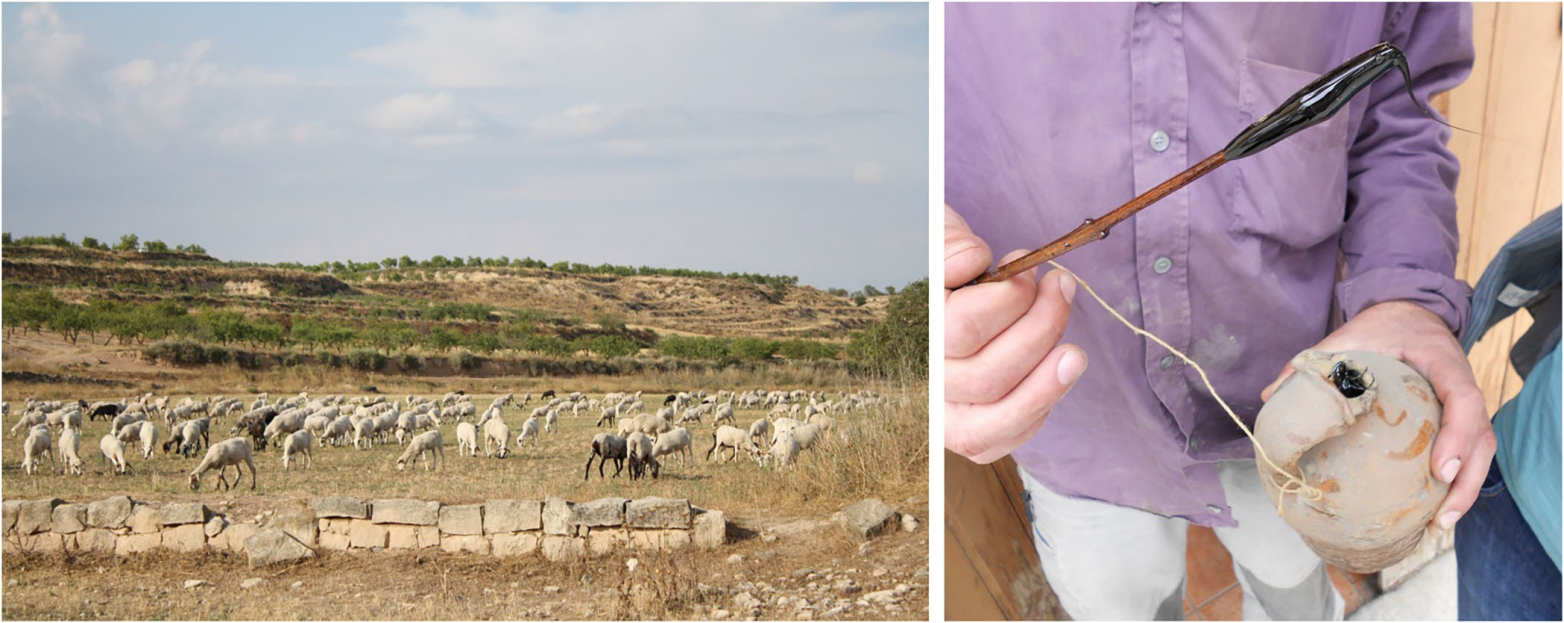
Sheep grazing and juniper oil (*Juniperus communis*) used for sheep diarrhea. Both pictures come from the ethnobotanical prospection in one of the studied territories.

Veterinary uses reported are less addressed to livestock than to home animals, namely, pets. Some uses are related to a specific animal group, such as the ruminant antistatic, which is exclusive of ruminants (in the studied territory, bovine, ovine, and caprine) or the anti-tympanic, a specific use addressed to prevent or to treat bovine tympanism, a gastrointestinal disease produced sometimes after consumption of pastures which include members of the Fabaceae family. A few other uses, such as for gaining weight or orexigenic, are generally applied to the livestock mostly to have healthy and well-fed animals and thus prevent potential health issues to arise. A curious example of one antidiarrheal application reported in the studied area is the use of a piece of cloth or wood soaked with juniper oil (*Juniperus communis* L.). Once soaked, the cloth is offered to the sheep, hoping that while being chewed up, part of the oil is released and ingested, helping to improve and/or stop diarrhea. The juniper oil (Figure 2) is obtained by dry distillation of the aerial parts of the plant. This oil is also commonly used to treat animal topical diseases, and in some cases, for human medicinal purposes as well.

The index of medicinal importance (MI) was also calculated for all the veterinary uses, and the results ranged from 1.00 to 13.15 (Table 2). The highest MI index corresponded to the purgative use (13.15), followed by those related to weight gaining (9.67), digestive (7.42), ruminant antistatic (7.38), carminative (7.22), and diaphoretic (7.00). The most quoted use, antidiarrheal, is not among the highest values, most likely because the diversity or the taxa reported for this use is elevated (56 taxa).

Regarding the pharmaceutical forms, 92.03% of the reports were related to applications of internal use, 6.90% to external use, and 1.07% were not identified. Among the 22 pharmaceutical forms recorded, tisane, including decoction and infusion, represented 58.69% of the total forms reported, followed by internal or external direct use (21.77%). Overall, these values are coincidental with those reported from general ethnoveterinary (and general ethnobotanical) works (9, 16), as well as with the only previous study on plants for gastrointestinal disorders in animals available in the bibliography that we are aware of (25).

### Pharmacological comparison

For each of the most quoted plants, an extensive bibliographic search was performed. Consistently, we focused on the specific uses claimed by the informants in both human and veterinary monographs from official sources and encyclopedic bibliography on phytotherapy detailed in Material and Methods section (Table 1). A total of 33.33% of the uses were confirmed in this literature search, which allows us to further comment on several aspects. First, the relevant number of plant popular uses in animal medicine that have been confirmed in pharmacological literature are promising candidates for future research focused on veterinary drug development. Second, the ethnoveterinary uses not detected in the pharmacological literature consulted, also open a door for further research on phytochemical and pharmacological studies on the plants concerned, either to confirm or discard the utility of popular knowledge. Besides this, many folk data on veterinary properties were confirmed in human phytotherapeutic or pharmacological sources, and these facts account for important similarities between remedies used to treat humans and animals, which is not surprising given the animal category to which human beings belong. Finally, we think it is worth highlighting the existence of an important caveat regarding the existence of comprehensive literature addressed to understanding the chemistry and pharmacological implications of many plants in veterinary sciences. Only cultures with a robust and longstanding traditional medicine background have some relevant bibliographic sources in the field, such as the Chinese (57), but in general, the coincidence with plants from Mediterranean areas is relatively scarce. The European Medicines Agency (52) includes one section specialized in dealing with veterinary, but not specifically related to plant-based applications, that is, phytotherapy. In any case, we are convinced that research aimed at identifying chemical compounds as well as the properties of plants used in ethnoveterinary should be encouraged, as it is done to a larger extent with plants used in traditional human medicine.

## Concluding remarks

The global corpus of plant-based ethnoveterinary knowledge for the gastrointestinal, metabolic, and nutritional disorders in the Catalan linguistic area is diverse. The present study compiled a high number of data for 148 taxa used to treat gastrointestinal disorders. The dataset is robust, as suggested by an informant consensus factor very close to 1. The ethnobotanicity index indicates that 3.35% of the Catalan flora is used to treat these types of disorders. The most reported taxon has been *Tanacetum parthenium* and the most common uses have been antidiarrheal, digestive, and laxative. Some reported uses are confirmed in encyclopedic pharmacological works, although still few of these works are specifically devoted to veterinary uses. To summarize, the results of the present study are relevant and should be taken into account, together with information from similar works in other geographical and cultural areas -which should be encouraged-, in research on new drugs for animal health.

## Data availability statement

The original contributions presented in the study are included in the article/Supplementary material, further inquiries can be directed to the corresponding author.

## Ethics statement

The studies involving human participants -in the present case not as patients, but as informants- were reviewed and approved. The information has been collected through semi-structured ethnobotanical interviews following the ethical principles of the International Society of Ethnobiology. The participants provided their informed consent to participate in this study. Written informed consent for participation was not required for this study in accordance with the national legislation and the institutional requirements.

## Author contributions

The subject and its reach have been designed by FC, JV and AG, with the assistance of the remaining authors on different points. AG and MP performed the database work to select and treat the ethnobotanical information of the areas chosen. FC, TG, and AG carried out the statistical analyzes. FC, JV, and AG wrote a version of the manuscript, which was read and discussed by all the authors and prepared the final version of the manuscript, which was read and approved by all the authors.

## Funding

This research was funded by projects 2017SGR001116 and CLT051/21/000005 from the Generalitat de Catalunya (Catalan Government), and PRO2020/2021/2022-S02-VALLES from the Institut d'Estudis Catalans (IEC, Catalan Academy of Sciences and Humanities). AG benefited from a postdoctoral contract of project CGL2017-84297-R of the Spanish government and a postdoctoral grant from the Universitat de Barcelona funded by NextGeneration EU funds (Margarita Salas 2022-2024).

## Conflict of interest

The authors declare that the research was conducted in the absence of any commercial or financial relationships that could be construed as a potential conflict of interest.

## Publisher's note

All claims expressed in this article are solely those of the authors and do not necessarily represent those of their affiliated organizations, or those of the publisher, the editors and the reviewers. Any product that may be evaluated in this article, or claim that may be made by its manufacturer, is not guaranteed or endorsed by the publisher.

## Footnotes

^1^Sáez L. *Bellaterra, Catalonia, Spain: Universitat Autònoma de Barcelona*. Personal communication. (2019).
